# Supplementary material for: High Levels of Low-Density Lipoproteins Correlate with Improved Survival in Patients with Squamous Cell Carcinoma of the Head and Neck
Source: Biomedicines. 2021 May 4;9(5):506. doi: 10.3390/biomedicines9050506 (PMC8147766; doi:10.3390/biomedicines9050506)
Supplement: Supplementary file 1 [file biomedicines-09-00506-s001.zip › biomedicines-1196155-supplementary.pdf]

## Supplementary information

A B C

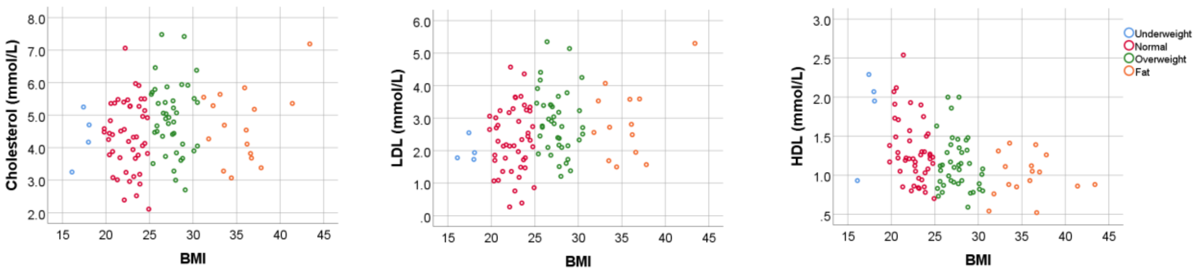


Correlation Coefficient = 0.132
*p* = 0.177

Correlation Coefficient = 0.217
*p* = 0.029

Correlation Coefficient = -0.380
*p* < 0.0001

**Figure. S1**. Correlations between body mass index (BMI) and lipoprotein levels. (A) Correlation between BMI and total cholesterol. (B) Correlation between BMI and low-density lipoprotein (LDL). (C) Correlation between BMI and high-density lipoprotein (HDL). Correlation coefficient and *p*-values are shown under the scatter plots.

A B

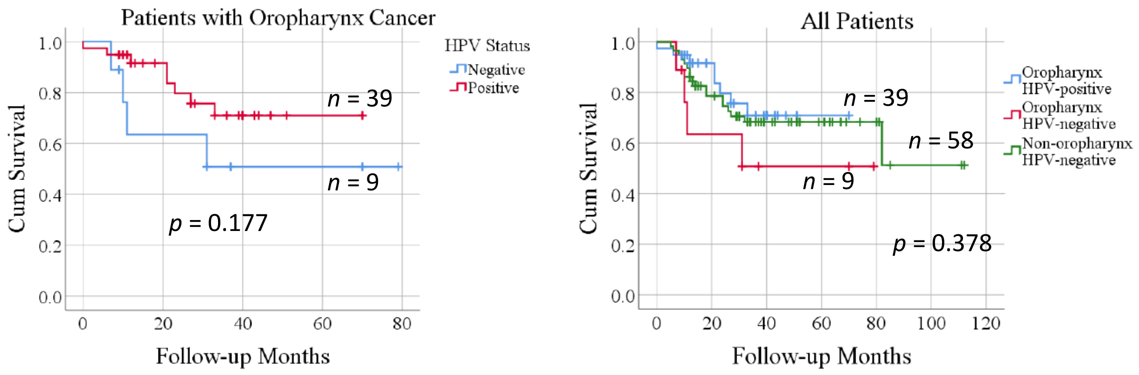


**Figure. S2**. Kaplan–Meier curves showing the impact of HPV status on overall survival. (A) Overall survival status between patients with HPV-negative and HPV-positive oropharynx cancer. (B) Overall survival status among patients with HPV-positive oropharynx, HPV-negative oropharynx or HPV-negative non-oropharynx cancers.
